# Time to restrict the use of p-values in *Acta Orthopaedica*

**DOI:** 10.1080/17453674.2018.1536526

**Published:** 2018-11-26

**Authors:** Jonas Ranstam

Several scientific journals have banned p-values, not because p-values are bad when used correctly, but because they are notoriously misused (Wasserstein and Lazar 2016).

## What is the problem with the p-value?

The problem with the p-value is not the p-value itself. The problem is ignorance about statistical inference, i.e., about the principles for using empirical observations and statistical reasoning to arrive at scientifically sound conclusions. An overwhelming majority of the authors of manuscripts submitted to medical journals believe, or at least seem to believe, that the p-value is a descriptive measure of importance regarding some aspect of an analyzed dataset: the lower the p-value, the stronger the effect. The truth of the matter is, however, that the p-value measures neither effect nor importance; it measures uncertainty. P-values are developed for performing rational generalization of findings, not for describing data.

Statistical hypothesis testing is performed on the basis of a statistical (null) hypothesis that specifies the properties of the population from which the studied data are collected and to which the generalization is made. The p-value is defined as the probability of drawing a random sample from this population being at least as unlikely as the observed one, given that the null hypothesis is true. For example, a p-value of 0.05 implies that the probability of drawing such a random sample is 1 in 20, or 5%, if the null hypothesis is correct. It is then a reasonable conclusion that the data and the null hypothesis are incompatible.

It is a common misunderstanding that the p-value indicates the size or importance of an observed effect. This is not the case. Whether or not a p-value is clinically important depends on the importance of the null hypothesis, not on the size of the p-value. Furthermore, as an example of random variation, while a major beneficial effect of a treatment in a population of patients with a specific disease may turn out to be statistically non-significant in an observed series of consecutive patients, a less effective treatment can be statistically significant.

It is also often believed that the p-value indicates something about the truth of the null hypothesis, or about the probability that the observed data have been caused by chance alone. Again, this is not the case. The p-value is simply based on the assumption that the null hypothesis is true.

The problems are, however, even greater than what these misunderstandings reflect. The null hypothesis is almost always believed to be directly related to the author’s research question, and the relation is assumed to be so unambiguous and straightforward that the null hypothesis does not need to be presented, let alone scientifically motivated. The truth is that most, if not all, medical research questions include problems with far greater complexity than can be solved using a single null hypothesis.

The links between the research question and its answer need to be developed prior to the statistical analysis in the form of a study design, accounted for in the statistical analysis, and explained and motivated to the reader in the study report. This represents a major intellectual challenge and is too often ignored or inadequately performed.

Instead, it is not uncommon for manuscripts to present hundreds of unstructured p-values in order to find the answer to the research question. These p-values do not all represent equally relevant null hypotheses, and already with a small number of hypothesis tests, the effects of multiplicity issues have deleterious consequences for the reliability of the tests’ outcome.

Furthermore, even in the apparently simple situation of comparing 2 groups of patients with respect to the mean value of 1 specific variable, many different null hypotheses can be defined: 3 traditional null hypotheses (1 2-sided and 2 1-sided, 1 in each direction), a number of 1-sided non-inferiority null hypotheses differing with respect to their non-inferiority margin, and a number of 2-sided equivalence null hypotheses differing with respect to their equivalence margin. Just testing 1 of these null hypotheses without motivating it may, perhaps, seem objective but is in fact subjective and has the potential to mislead the reader.

One consequence of the p-value misunderstandings and ignorance of statistical inference is the dichotomization of findings as either “significant” or “not significant” without any consideration of what is tested and of the risk of false positive and negative outcomes. The interpretation of these 2 categories as indicating “important” and “no difference” is wide off the mark. Whether a specific p-value can be interpreted as indicating importance depends on several things, among them the medical or biological relevance of the research question, the soundness of the study design, and the definition of the null hypothesis. It takes more than a “p < 0.05” to show scientific importance, and “p > 0.05” is not an indication of “no difference”; it reflects absence of evidence but not evidence of absence.

The misunderstandings are, unfortunately, ubiquitous in medical research. It is not hard to find examples of this: 3 are presented below.

## Example 1. Confusing sample and population

The aim of a study is described as to study a general phenomenon, e.g., “to find risk factors for hip fracture among subjects older than 65 years of age.” However, instead of generalizing the findings to all subjects over 65, the author’s conclusion is merely a brief description of what has been observed in his hospital series, referring to statistical significance as an indication of importance, e.g., “among the studied patients, smoking was a significant risk factor.” However, the null hypotheses underlying the presented p-values are all about the entire population of subjects over 65, including future patients, not about the studied hospital series.

## Example 2. Testing an irrelevant null hypothesis

In a matched case-control study, p-values are used for evaluating the pairwise differences in matching variables between matched cases and controls. The tests are performed with the confused purpose to assess whether the observed differences of matched cases and controls are clinically important. The underlying null hypotheses, however, do not refer to the observed cases and controls but to the infinite population represented by the matched cases and controls, and for this matching is not a relevant issue.

## Example 3. Testing baseline imbalance after randomization

P-values are wrongly used for evaluating baseline imbalance after randomization in a clinical trial. The purpose is to investigate if the randomization “was successful”, i.e., resulted in groups with the same baseline characteristics. Randomization is, however, used to prevent systematic errors in the generalization of the trial’s results (what the outcome means for patients in general), not to eliminate random imbalance among the randomized patients. Random imbalance of known prognostic factors can be avoided by stratifying the randomization on these factors.

Many more examples can be presented, but the ones above should be sufficient to describe the presented phenomena.

## Can we just skip the p-value?

Yes, and orthopedic research would benefit from it. Confidence intervals represent a superior way to present generalization uncertainty. Confidence intervals have the advantage of measuring the uncertainty of the size of an estimated effect, which p-values do not. And in contrast to when using p-values, questions regarding the clinical significance of, and empirical support for, a specific conclusion can be directly answered by the effect sizes that are included in, or excluded from, a confidence interval.

## 2 simple principles

To start a transition to p-value-free manuscripts, *Acta Orthopaedica* will enforce a policy of zero tolerance vis-à-vis p-value misconceptions. Authors who wish to publish manuscript with p-values must from now on comply with 2 principles for concluding whether or not scientifically important differences exist:

1. A statistically non-significant test is not sufficient to claim “no difference.” To show “no difference,” a smallest clinically relevant size of the difference (it might be 0) must be defined. If all clinically relevant differences are excluded from the difference’s 95% confidence interval a “no difference” conclusion is reasonable.
2. A statistically significant test is not necessarily related to a clinically important difference. The importance of the tested null hypothesis must be motivated using other arguments than the p-value, and a smallest clinically relevant difference (it might be 0) must be defined, and if the difference’s 95% confidence interval excludes all clinically irrelevant differences, a conclusion about the existence of a clinically important difference is reasonable.

The required clinical definitions, motivations, and explanations should be presented in the manuscript in non-technical terms. For example (from Paavola et al. 2018), the null hypothesis and clinically relevant difference can be described: “Main outcome measure: Shoulder pain at rest … (visual analogue scale (VAS) from 0 to 100, with 0 denoting no pain), at 24 months. The threshold for minimal clinically important difference was set at 15.” The result is presented as: “In the primary intention to treat analysis (ASD versus diagnostic arthroscopy), no clinically relevant between group differences were seen… The … difference between groups … in pain VAS was −5 (95% confidence interval −11 to 2) points.”

The above example shows that confidence intervals are better than p-values, and that p-values are redundant when confidence intervals are presented. Arguments against replacing p-values with confidence intervals are usually motivated by a desire to be able to keep misusing p-values for showing “no difference” and “importance” without having to consider clinical relevance and without having to argue in clinical terms why an observed difference is important. The failure of such simplistic research is, however, clearly shown in the discussion on the reproducibility crisis of modern biomedical research. It is now time for a more serious approach.
